# Duality of Shehu transform with other well known transforms and application to fractional order differential equations

**DOI:** 10.1371/journal.pone.0318157

**Published:** 2025-04-29

**Authors:** Nabil Mlaiki, Noor Jamal, Muhammad Sarwar, Manel Hleili, Khursheed J. Ansari

**Affiliations:** 1 Department of Mathematics and General Sciences, Prince Sultan University, Riyadh, Saudi Arabia; 2 Department of Mathematics, University of Malakand, Chakdara Dir (Lower), Khyber Pakhtunkhawa, Pakistan; 3 Department of Mathematics, Faculty of Science, University of Tabuk, Tabuk 71491 Saudi Arabia; 4 Department of Mathematics, College of Science, King Khalid University, Abha, Saudi Arabia; Sefako Makgatho Health Sciences University Faculty of Health Sciences, SOUTH AFRICA

## Abstract

Integral transforms are used in many research articles in the literature, due to their interesting applications in the solutions of problems of applied science and engineering. In many situations, researchers feel difficulties in applying a given transform to solve differential or integral equations, therefore it is more convenient to derive dualities relations between these transforms. Shehu transform has the properties to converge to the well-known integral transforms used in the literature only by changing the space parameters. In this article, we will derive the inter-conversion relations between the Shehu transform, Natural, Sumudu, Laplace, Laplace-Carson, Fourier, Aboodh, Elzaki, Kamal and Mellin transforms. These duality relations will make simple the integral transforms because if a transform such as Fourier or Mellin transform is difficult to solve a differential equation due to its complexity then duality relations will do this job easily. These multiplicity relations have many interesting properties that make visualizations easier. The duality relations have important applications in solving the fractional order differential equations by various integral transforms. Moreover, duality relations save the time of researchers, because in the literature the researchers solved a problem with different transforms. Keeping in mind these advantages of the duality relations, we decide to discuss the duality relations of Shehu transform with other integral transforms.

## 1 Introduction

Integral transforms are widely used to solve differential equations because they can simplify the process by converting complex differential equations into simpler algebraic equations. Algebraic equations are generally easier to manipulate and solve. Integral transforms can effectively handle initial and boundary conditions. It incorporates initial conditions directly into the transformed equations, which simplifies the process of finding the solutions. Integral transforms are particularly effective for linear differential equations, as they preserve the linearity of the system. Moreover, in many applications, such as systems with memory or delay, the differential equations involve convolution integrals. The integral transforms convert convolution into simple multiplication, making it easier to handle these terms. Therefore, in many research articles in the literature, one can find integral transforms and their applications by solving many problems of applied science and engineering. The differential equations can be solved by different integral transforms.

The commonly used integral transforms are Laplace [1], Fourier [2], Mellin [3], Laplace-Carson [4], Sumudu [5], natural [6], Elzaki [7], Aboodh [8], Kamal [9] and Shehu transform [10]. The ancestor of integral transforms is the work of Laplace in 1780, Fourier in 1822, Mellin and John Renshaw Carson. Watugaia introduced Sumudu transform [5] in 1993 and solved differential equations and control engineering problems in [11]. Belgacem et al. [11] study applications of sumudu transform in integral equations. Sumudu transform is used to solve partial differential equations in [12] and [13], while for fractional differential equations in [14]. The authors of papers [15,16] used Laplace Optimized Decomposition Method for the solutions of fractional order differential equations. Khan and Khan [6] in 2008, established natural transform and first applied it to the solutions of fluid problems and then to Maxwell’s equations. The authors [17] and [18] treat Maxwell’s equations with natural transform. Natural transform is used to solve differential equations in [19] and fractional differential equations [20]. A new transform known as the Shehu transform was introduced by Shehu and Weldong [10] for solving both ordinary and partial differential equations. Shehu transform has interested applications for the solutions of fractional order linear and non-linear differential equations see [21–27].

A natural question arises, can these integral transforms be related to each other? To answer this question, Khalaf et al. [28] studied the dualities of the Sumudu transform with, Mellin, Fourier and Laplace transforms. Shah et al. [29] extracted Laplace, Sumudu, Fourier and Mellin transform from natural transform. Chauhan et al. [30] extracted Laplace, Sumudu, Elzaki, Kamal, and Aboodh transform from Laplace-Carson. In many situations, it is very difficult to apply an integral transform to a problem directly but the duality relation is easily applicable. For example, if the direct use of the Mellin transform to solve differential or integral equations is difficult, then it is more convenient to use the duality relation of the Mellin transform with other integral transforms. The duality relation makes many applied problems of science and technology simpler to solve. The many interesting properties of the duality relations that make visualizations easier, motivate us to take an interest in obtaining the duality relations between the Shehu transform and other integral transforms.

In this article, we will derive dualities relations between the Shehu transform and other transforms (natural, Sumudu, Laplace, Laplace-Carson, Fourier, Aboodh, Elzaki, Kamal, Mellin transforms). Shehu transform has the properties to converge to these transforms only by changing the space parameters. We will derive links among these transforms to show that the Shehu transform acts as a source to inter-convert these integral transforms into one another. We will derive some interesting inter-conversion relations between Shehu and another transform. These duality relations can be utilized to solve many complicated problems of fluid mechanics and other scientific disciplines like Physics, Chemistry, dynamics etc. Moreover, these duality relations will save the time of researchers, because in the literature the researchers solved a problem with different transforms. These duality relations will directly provide us results of all transforms of a problem solved by a single transform. Moreover, we are interesting to derive the duality relations of papers [28–30] from this study. Furthermore, we will discuss the applications of these duality relations for the solutions of fractional order differential equations. For the authenticity of this work, we will provide examples, once we solve it by Shehu, Natural, Sumudu, Laplace, Laplace-Carson, Fourier, Elzaki, Kamal, Aboodh, Mellin transform directly and then with duality relations.

## 2 Preliminaries related to Shehu transform and transform of fractional orderdifferential equations

** Definition 2.1. **[10]. The Shehu transform of the function *f*(*t*) of exponential order is defined as follows


$$\begin{array}{ccc} S\{f(t)\} & =\int_{0}^{\infty }e^{\frac{-st}{u}}f(t)dt, & \end{array}$$ (1)

V^(s,u)= lim ⁡ α→∞∫ 0αe−stuf(t)dt,s,u>0. (2)

where, *f*(*t*) is function that satisfy the condition of exponential order meaning that there exist $N,p_{1},p_{2}>0$ such that $|f(t)|<Ne^{(\frac{\left|t\right|}{p_{i}})}$ for all $t\in {\left(-1\right)}^{i}\times [0,\infty ).$ This condition ensures that the function does not grow faster than an exponential function, allowing the integral to converge. The set of functions satisfy the condition of exponential order is define as


$$\begin{array}{ccc} V=\{f(t):\exists \;\;N,p_{1},p_{2}>0,|f(t)|<Ne^{(\frac{\left|t\right|}{p_{i}})},if\;\;t\in {\left(-1\right)}^{i}\times [0,\infty )\}. & & \end{array}$$ (3)

Inverse transform can be define as $S^{-1}(\hat{V}(s,u))=f(t),$ for *t* ≥ 0 ,  this is equivalent to


$$\begin{array}{ccc} f(t)=S^{-1}(\hat{V}(s,u))=\frac{1}{2\pi i}\int_{\alpha -i\infty }^{\alpha +i\infty }\frac{1}{u}e^{\frac{-s}{u}t}\hat{V}(s,u)ds. & & \end{array}$$ (4)

Here *s* and *u* are transform variables and integral is taken along *s* = *α* in the complex plane *s* = *x* + *iy* where *α* is real constant.

** Definition 2.2 (**Linearity property** [10]). **Let functions *g* ( *t* ) , *f* ( *t* ) ∈ *V* then for arbitrary non-zero constants *a* and *b* ,  *ag* ( *t* ) + *bf* ( *t* ) ∈ *V* and *S* { *ag* ( *t* ) + *bf* ( *t* ) } = *aS* { *g* ( *t* ) } + *bS* { *f* ( *t* ) } . 

** Definition 2.3 (**Change of scale property** [10]). **Let functions *f* ( *αt* ) ∈ *V* for arbitrary non-zero constants *α* ,  then $S\{f(\alpha t)\}=\frac{u}{\alpha }\hat{V}(\frac{s}{\alpha },u).$

** Definition 2.4 (**Sufficient condition for the existence of Shehu Transform** [10]). **Let function *f*(*t*) is of exponential order *a* for *t* > *b* and continues piecewise in each interval 0 ≤ *t* ≤ *b* .  Then Shehu transform $\hat{V}(s,u)$ exists.

** Theorem 2.5. **[26] The necessary condition for the function $\hat{V}(s,u)$ to be the Shehu transform of generalized function *f*(*t*) are that $\hat{V}(s,u)$ is analytic on *V* and for each closed strip $\left\{u:a\leq Re(\frac{s}{u})\leq b\right\}$ of *V* there be polynomial such that $\left|\hat{V}(s,u)|\leq P(|\frac{s}{u}|\right)$ for $a\leq Re(\frac{s}{u})\leq b.$ The polynomial *P* will depends in general on *a* and *b*.

** Theorem 2.6. **[26] (**Inverse Shehu Transform** Let $\hat{V}(s,u)=S\{f(t)\}$ for $r_{1}<Re(\frac{s}{u})<r_{2}$, let *q* be any real variable then in the sense of convergence in *V* . 

f(t)= lim ⁡ p→∞12πi ∫  ⁡ r−ipr+ipe−sutV^(s,u)ds. Where *r* is fixed number such that $r_{1}<r<r_{2}s=r+iw$.

** Theorem 2.7. ** [10] If the function *y*(*t*) has nth order derivative $y^{n}(t)$ then its Shehu transform is define as


$$\begin{array}{ccc} S(y^{n}(t))=\frac{s^{n}}{u^{n}}\hat{V}(s,u)-\overset{n-1}{\underset{k=0}{\sum }}(\frac{s}{u}{)}^{n-(k+1)}y^{k}(0),n\geq 0. & & \end{array}$$ (5)

** Theorem 2.8. ** [31] If the function *y*(*t*) has nth order derivative $y^{n}(t)$ then its Shehu transform is define as


$$\begin{array}{ccc} N(y^{n}(t))=\frac{s^{n}}{u^{n}}R(s,u)-\overset{n-1}{\underset{k=0}{\sum }}\frac{s^{n-(k+1)}}{u^{n-k}}y^{k}(0),n\geq 0. & & \end{array}$$ (6)

** Definition 2.9. **[32] Let suppose $y(x)\in L_{1}(a,b)$, the caputo fractional order derivative of order *n* − 1 < *α* ≤ *n* is define by


$$\begin{array}{c} {\;}^{c}D_{0^{+}}^{\alpha }y(x)=\{\begin{array}{c} \frac{1}{\Gamma (n-\alpha )}\int_{0}^{x}{\left(x-\chi \right)}^{n-\alpha -1}y^{n}(\chi )d\chi ,n-1<\alpha \leq n,\\ \frac{\partial^{n}}{\partial x^{n}}y(x),if\;\;n=\alpha .\; \end{array} \end{array}$$ (7)

** Theorem 2.10. ** [33] Let functions $y(t)\in AC^{n}(a,b)$ where *a* < *b* and also *y* ( *t* ) ∈ *V* then Shehu transform of caputo fractional order derivative is given as


$$\begin{array}{ccc} S{(}^{c}D_{0}^{\alpha }y(t))=(\frac{s}{u}{)}^{\alpha }\hat{V}(s,u)-\overset{n-1}{\underset{k=0}{\sum }}(\frac{s}{u}{)}^{\alpha -(k+1)}y^{k}(0),where\;\;n-1\leq \alpha <n. & & \end{array}$$ (8)

** Definition 2.11. ** [34] The Laplace transform of Caputo fractional order derivative of functions *y*(*t*) is define as


$$\begin{array}{ccc} L{(}^{c}D_{0}^{\alpha }y(t))={\left(s\right)}^{\alpha }F(s)-\overset{n-1}{\underset{k=0}{\sum }}{\left(s\right)}^{\alpha -(k+1)}y^{k}(0),where\;\;n-1\leq \alpha <n. & & \end{array}$$ (9)

** Theorem 2.12. ** [35] The sumudu transform of caputo fractional order derivative of functions *y* ( *t* ) ,  of order *α* where *n* − 1 ≤ *α* < *n* is given as


$$\begin{array}{ccc} \hat{S}{(}^{c}D_{0}^{\alpha }y(t))={\left(u\right)}^{-\alpha }\{G(u)-\overset{n-1}{\underset{k=0}{\sum }}{\left(u\right)}^{k}y^{k}(0)\}. & & \end{array}$$ (10)

** Proposition 2.1. ** [20] The natural transform of caputo fractional order derivative of functions *y*(*t*) is define as


$$\begin{array}{ccc} N{(}^{c}D_{0}^{\alpha }y(t))=(\frac{s}{u}{)}^{\alpha }R(s,u)-\overset{n-1}{\underset{k=0}{\sum }}\frac{s^{\alpha -(k+1)}}{u^{\alpha -k}}y^{k}(0),where\;\;n-1\leq \alpha <n. & & \end{array}$$ (11)

## 3 Duality relations between Shehu transform and other transforms

In this section, we will discuss the duality relations between Shehu transform and other transforms. We will also provide examples for the usability of the established relations.

**Duality between Shehu and Natural Transform**

The natural transform of the function *f*(*t*) is *R* ( *s* , *u* )  given by, $R(s,u)=N\{f(t)\}=\int_{0}^{\infty }e^{-st}f(ut)dt.$

** Theorem 3.1. ** Let the function *f* ( *t* ) ∈ *V* has Shehu transform $S\{f(t)\}=\hat{V}(s,u)$ and natural transform *N* { *f* ( *t* ) } = *R* ( *s* , *u* )  then $\hat{V}(s,u)=uR(\frac{s}{u},u)$ and $R(s,u)=\frac{1}{u}V(us,u).$

** Proof. **Since Shehu transform is define as $\hat{V}(s,u)=S(f(t))=\int_{0}^{\infty }e^{\frac{-st}{u}}f(t)dt,$ by substituting $t=uw\Rightarrow \frac{t}{u}=w$ and *dt* = *udw* ,  then we have


$$\begin{array}{cccc} \hat{V}(s,u) & =S(f(t))=\int_{0}^{\infty }e^{-sw}f(uw)udw=uR(h,u),where\;\;h=\frac{s}{u} & & \\ \hat{V}(s,u) & =uR(\frac{s}{u},u). & \end{array}$$ (12)

Now, by definition of natural transform, we have $R(s,u)=N\{f(t)\}=\int_{0}^{\infty }e^{-st}f(ut)dt.$ By substituting, $ut=w\Rightarrow t=\frac{w}{u}$ and *udt* = *dw* ,  then we have


$$\begin{array}{ccc} R(s,u) & = & \int_{0}^{\infty }e^{-st}f(ut)dt=\int_{0}^{\infty }e^{\frac{-s}{u}w}f(w)\frac{dw}{u}=\frac{1}{u}\int_{0}^{\infty }e^{\frac{-s}{u}w}f(w)dw, \end{array}$$ (13)

$$\begin{array}{ccc} R(s,u) & = & \frac{1}{u}\hat{V}(k,u)=\frac{1}{u}\hat{V}(su,u)\;where\;\;k=su. \end{array}$$ (14)

□

** Example 1. **Consider, $f(t)=\frac{t^{2}}{2!}$ and $g(t)=e^{3t},$ then natural and Shehu Transform are given by


$$\begin{array}{cc} R(s,u)=N\{\frac{t^{2}}{2!}\}=\frac{u^{2}}{s^{3}}\;\;and\;\;\hat{V}(s,u)=S\{\frac{t^{2}}{2!}\}=(\frac{u}{s}{)}^{3}=u\frac{u^{2}}{s^{3}}\Rightarrow \hat{V}(s,u)=uR(s,u). & \end{array}$$

Also


$$\begin{array}{cc} R(s,u)=N\{e^{3t}\}=\frac{1}{s-3u}\;\;and\;\;\hat{V}(s,u)=S\{e^{3t}\}=\frac{u}{s-3u}\Rightarrow \hat{V}(s,u)=uR(s,u). & \end{array}$$

**Duality between Shehu and Laplace Transform**

Let *F* ( *s* ) ,  is Laplace transform of a function *f* ( *t* ) ,  define by $F(s)=L\{f(t)\}=\int_{0}^{\infty }e^{-st}f(t)dt.$

** Theorem 3.2. ** Let the function *f* ( *t* ) ∈ *V* ,  has Shehu transform $S\{f(t)\}=\hat{V}(s,u)$ and Laplace transform *L* { *f* ( *t* ) } = *F* ( *s* )  then $\hat{V}(s,u)=F(\frac{s}{u})$ and $F(s)=\hat{V}(su,u)$

** Proof. **Since Shehu transform is define by $\hat{V}(s,u)=S(f(t))=\int_{0}^{\infty }e^{\frac{-s}{u}t}f(t)dt$ by substituting, $\frac{s}{u}=w$ then, we have


$$\begin{array}{cccc} \hat{V}(s,u) & =\int_{0}^{\infty }e^{\frac{-s}{u}t}f(t)dt=\int_{0}^{\infty }e^{-wt}f(t)dt=F(w) & & \\ \hat{V}(s,u) & =F(w)=F(\frac{s}{u}). & \end{array}$$ (15)

Now from Laplace transform one can get, $F(s)=L\{f(t)\}=\int_{0}^{\infty }e^{-st}f(t)dt.$ By substituting $s=\frac{w}{u}$ then, we have


$$\begin{array}{ccc} F(s) & =\int_{0}^{\infty }e^{-st}f(t)dt=\int_{0}^{\infty }e^{\frac{-w}{u}t}f(t)dt=\hat{V}(w,u), & \end{array}$$ (16)

$$\begin{array}{ccc} F(s) & =\hat{V}(w,u)=\hat{V}(su,u). & \end{array}$$ (17)

□

** Example 2. **Let $f(t)=\frac{\sin(2t)}{2}+\cos(3t)$ then take natural transform *F*(*s*) and Shehu Transform $\hat{V}(su,u)$ is follows


$$\begin{array}{cc} F(s)=L\{\frac{\sin(2t)}{2}+\cos(3t)\}=\frac{1}{s^{2}+4}+\frac{s}{s^{2}+4}\;\;and & \end{array}$$

$$\begin{array}{cc} \hat{V}(su,u)=S\{\frac{\sin(2t)}{2}+\cos(3t)\}=\frac{u^{2}}{{\left(us\right)}^{2}+4u^{2}}+\frac{u^{2}s}{{\left(us\right)}^{2}+4u^{2}}=\frac{1}{s^{2}+4}+\frac{s}{s^{2}+4}=F(s). & \end{array}$$

Now, take natural transform $F(\frac{s}{u})$ and Shehu transform $\hat{V}(s,u)$ of the function $f(t)=\frac{\sin(2t)}{2}+\cos(3t),$ is follows


$$\begin{array}{cc} F(\frac{s}{u})=L\{\frac{\sin(2t)}{2}+\cos(3t)\}=\frac{1}{(\frac{s}{u}{)}^{2}+4}+\frac{(\frac{s}{u})}{(\frac{s}{u}{)}^{2}+4}=\frac{u^{2}}{s^{2}+4u^{2}}+\frac{us}{s^{2}+4u^{2}}\;\;and & \\ \hat{V}(s,u)=S\{\frac{\sin(2t)}{2}+\cos(3t)\}=\frac{u^{2}}{s^{2}+4u^{2}}+\frac{us}{s^{2}+4u^{2}}=F(\frac{s}{u}). & \end{array}$$

**Duality between Shehu and Sumudu Transform** Let *G*(*s*) is Sumudu transform of a function *f*(*t*) define by $G(s)=\hat{S}\{f(t)\}=\int_{0}^{\infty }e^{-t}f(ut)dt.$

** Theorem 3.3. ** Let function *f* ( *t* ) ∈ *V* has Shehu transform $S\{f(t)\}=\hat{V}(s,u)$ and Sumudu transform $\hat{S}\{f(t)\}=G(u)$ then $\hat{V}(s,u)=\frac{u}{s}G(\frac{u}{s})$ and $G(u)=\frac{s}{u}\hat{V}(s,u).$

** Proof. **Since Shehu transform is define as, $\hat{V}(s,u)=S(f(t))=\int_{0}^{\infty }e^{\frac{-st}{u}}f(t)dt,$ by substituting, $\frac{st}{u}=w\Rightarrow t=\frac{uw}{s}$ and $dt=\frac{u}{s}dw,$ then we have


$$\begin{array}{cccc} \hat{V}(s,u) & =\int_{0}^{\infty }e^{\frac{-s}{u}t}f(t)dt=\int_{0}^{\infty }e^{-w}f(\frac{uw}{s})\frac{u}{s}dw=\frac{u}{s}\int_{0}^{\infty }e^{-w}f(\frac{uw}{s})dw & & \\ \hat{V}(s,u) & =\frac{u}{s}G(\frac{u}{s}). & \end{array}$$ (18)

Now, take Sumudu transform function *f*(*t*) as follows, $G(s)=\hat{S}\{f(t)\}=\int_{0}^{\infty }e^{-t}f(st)dt.$ By substituting $t=\frac{sw}{u}\Rightarrow dt=\frac{s}{u}dw,$ then we have


$$\begin{array}{ccc} G(u) & =\int_{0}^{\infty }e^{-t}f(ut)dt=\int_{0}^{\infty }e^{-\frac{s}{u}w}f(sw)\frac{s}{u}dw=\frac{s}{u}\int_{0}^{\infty }e^{-\frac{s}{u}w}f(sw)dw, & \end{array}$$ (19)

$$\begin{array}{ccc} G(u) & =\frac{s}{u}\hat{V}(s,u). & \end{array}$$ (20)

□

** Example 3. **Consider $f(t)=\frac{\sin(6t)}{6}+\cos(4t),$ then Sumudu transform $G(\frac{u}{s})$ and Shehu transform are given by


$$\begin{array}{cc} G(\frac{u}{s})=\hat{S}\{\frac{\sin(6t)}{6}+\cos(4t)\}=\frac{\frac{u}{s}}{1+36(\frac{u}{s}{)}^{2}}+\frac{1}{1+16(\frac{u}{s}{)}^{2}}=\frac{us}{s^{2}+36u^{2}}+\frac{s^{2}}{s^{2}+16u^{2}}\;\;and & \end{array}$$

$$\begin{array}{cc} \hat{V}(s,u)=S\{\frac{\sin(6t)}{6}+\cos(4t)\}=\frac{u^{2}}{s^{2}+36u^{2}}+\frac{us}{s^{2}+16u^{2}}=\frac{u}{s}(\frac{us}{s^{2}+36u^{2}}+\frac{s^{2}}{s^{2}+16u^{2}})=\frac{u}{s}G(\frac{u}{s}). & \end{array}$$

**Duality between Shehu and Mellin Transform** Let *M*(*s*) is Mellin transform of the function *f* ( *t* ) ,  define by $M(s)=M\{f(t),s\}=\int_{0}^{\infty }t^{s-1}f(t)dt.$

** Theorem 3.4. ** Let *f* ( *t* ) ∈ *V* and $S\{f(t)\}=\hat{V}(s,u)$ and *M* ( *s* ) = *M* { *f* ( *t* ) , *s* }  then V^(s,u)=M{f(−ln ⁡ t),s} and $M(s)=-\frac{1}{u}S\{f(e^{-\frac{t}{u}})\}.$

** Proof. **Since $\hat{V}(s,u)=S(f(w))=\int_{0}^{\infty }e^{\frac{-s}{u}w}f(w)dw,$ by substituting, w=−ln ⁡ t⇒dw=−1tdt, then we have


V^(s,u)= ∫ 0∞e−suwf(w)dw= ∫ 0∞e(−su)(− ln ⁡ t)f(−ln ⁡ t)−1tdt=−∫ 0∞tsut−1f(−ln ⁡ t)dt (21)

V^(s,u)=−∫ 0∞tsu−1f(−ln ⁡ t)dt=M{f(−ln ⁡ t),su} (22)

V^(s,u)=M{f(−ln ⁡ t),su}. (23)

Now, the Mellin transform of *f*(*t*) is $M(s)=M\{f(w),s\}=\int_{0}^{\infty }w^{s-1}f(w)dw,$ by substituting $w=e^{-\frac{t}{u}}\Rightarrow dw=-\frac{1}{u}e^{\frac{-t}{u}}dt$ then, we have


$$\begin{array}{ccc} M(s) & =\int_{0}^{\infty }e^{-\frac{t}{u}(s-1)}f(e^{-\frac{t}{u}})(-\frac{1}{u})e^{\frac{-t}{u}}dt=-\frac{1}{u}\int_{0}^{\infty }e^{\frac{-s}{u}t}f(e^{-\frac{t}{u}})dt, & \end{array}$$ (24)

$$\begin{array}{ccc} M(s) & =-\frac{1}{u}S\{f(e^{-\frac{t}{u}})\}. & \end{array}$$ (25)

□

** Example 4. **Consider, *f* ( *t* ) = * sin* ⁡  ( *t* ) + * cos* ⁡  ( *t* ) ,  then Mellin and Shehu Transform are given by,


$$\begin{array}{cc} M(s)=M\{\sin(t)+\cos(t)\}=\Gamma (s)\{\sin(\frac{\Pi s}{2})+\cos(\frac{\Pi s}{2})\}\;\;and & \\ \hat{V}(s,u)=S\{\sin(t)+\cos(t)\}=\frac{u^{2}}{s^{2}+u^{2}}+\frac{us}{s^{2}+u^{2}}. & \end{array}$$

**Duality between Shehu and Laplace-Carson Transform**

Let ${\hat{f}}_{c}(p^{\prime }),$ is Laplace-Carson transform of a function *f* ( *t* ) ,  define by ${\hat{f}}_{c}(p^{\prime })=p^{\prime }\int_{0}^{\infty }e^{-p^{\prime }t}f(t)dt.$

** Theorem 3.5. ** Let function *f* ( *t* ) ∈ *V* has Shehu transform $S\{f(t)\}=\hat{V}(s,u)$ and Laplace-Carson transform ${\hat{f}}_{c}(p^{\prime })=C\{f(t)\},$ then $\hat{V}(s,u)=\frac{u}{s}{\hat{f}}_{c}(\frac{s}{u})$ and ${\hat{f}}_{c}(\frac{s}{u})=\frac{s}{u}\hat{V}(s,u).$

** Proof. **Since Shehu transform is define as $\hat{V}(s,u)=S(f(t))=\int_{0}^{\infty }e^{\frac{-s}{u}t}f(t)dt,$ by substituting, $\frac{s}{u}=p^{\prime }$ then, we have


$$\begin{array}{cccc} \hat{V}(s,u) & =S(f(t))=\int_{0}^{\infty }e^{-\frac{s}{u}t}f(t)dt=\frac{p^{\prime }}{p^{\prime }}\int_{0}^{\infty }e^{-p^{\prime }t}f(t)dt=\frac{1}{p^{\prime }}\int_{0}^{\infty }e^{-p^{\prime }t}f(t)dt=\frac{1}{p^{\prime }}{\hat{f}}_{c}(p^{\prime }). & & \\ \hat{V}(s,u) & =\frac{1}{p^{\prime }}{\hat{f}}_{c}(p^{\prime })=\frac{u}{s}{\hat{f}}_{c}(\frac{s}{u})\;where\;\;p^{\prime }=\frac{s}{u}. & \end{array}$$ (26)

Now, from definition of Laplace-Carson transform ${\hat{f}}_{c}(p^{\prime })=p^{\prime }\int_{0}^{\infty }e^{-p^{\prime }t}f(t)dt,$ by substituting, $p^{\prime }=\frac{s}{u}$ then, we have


$$\begin{array}{ccc} {\hat{f}}_{c}(p^{\prime }) & =C\{f(t)\}=p^{\prime }\int_{0}^{\infty }e^{-p^{\prime }t}f(t)dt=\frac{u}{s}\int_{0}^{\infty }e^{\frac{-s}{u}t}f(t)dt=\frac{s}{u}\hat{V}(s,u), & \end{array}$$ (27)

$$\begin{array}{ccc} {\hat{f}}_{c}(\frac{s}{u}) & =\frac{s}{u}\hat{V}(s,u)\;\;where\;\;p^{\prime }=\frac{s}{u}. & \end{array}$$ (28)

□

** Example 5. **Let us consider, *f* ( *t* ) = * sinh* ⁡  ( 2*t* ) + * cosh* ⁡  ( 3*t* ) ,  the Laplace-Carson transform ${\hat{f}}_{c}(\frac{s}{u})$ and Shehu transform $\hat{V}(s,u)$ are given by,


$$\begin{array}{cc} \hat{V}(s,u)=S(f(t))=S\{\sinh(2t)+\cosh(3t)\}=\frac{2u^{2}}{s^{2}-4u^{2}}+\frac{us}{s^{2}-9u^{2}}. & \end{array}$$

Now, apply Laplace-Carson transform as follows


$$\begin{array}{cc} {\hat{f}}_{c}(\frac{s}{u})=C\{\sinh(2t)+\cosh(3t)\}=\frac{2\frac{s}{u}}{(\frac{s}{u}{)}^{2}-4}+\frac{(\frac{s}{u}{)}^{2}}{(\frac{s}{u}{)}^{2}-9}=\frac{2su}{s^{2}-4u^{2}}+\frac{s^{2}}{s^{2}-9u^{2}} & \\ =\frac{s}{u}(\frac{2u^{2}}{s^{2}-4u^{2}}+\frac{us}{s^{2}-9u^{2}})=\frac{s}{u}\hat{V}(s,u). & \end{array}$$

**Duality between Shehu and Elzaki Transform**

Let *T*(*p*) is the Elzaki transform of the function *f*(*t*) define by $T(p)=E\{f(t)\}=p\int_{0}^{\infty }e^{\frac{-t}{p}}f(t)dt.$

** Theorem 3.6. ** Let function *f* ( *t* ) ∈ *V* has Shehu transform $S\{f(p)\}=\hat{V}(s,u)$ and Elzaki transform *T* ( *p* ) = *E* { *f* ( *t* ) } ,  then $\hat{V}(s,u)=\frac{s}{u}T(\frac{u}{s})$ and $T(\frac{u}{s})=\frac{u}{s}\hat{V}(s,u).$

** Proof. **Since Shehu transform of the function *f*(*t*) is define as, $\hat{V}(s,u)=S(f(t))=\int_{0}^{\infty }e^{\frac{-st}{u}}f(t)dt$ by substituting, $\frac{s}{u}=\frac{1}{p}$ then, we have


$$\begin{array}{cccc} \hat{V}(s,u) & =S(f(t))=\int_{0}^{\infty }e^{\frac{-s}{u}t}f(t)dt=\frac{p}{p}\int_{0}^{\infty }e^{\frac{-t}{p}}f(t)dt=\frac{1}{p}\int_{0}^{\infty }e^{\frac{-t}{p}}f(t)dt=\frac{1}{p}T(p) & & \\ \hat{V}(s,u) & =\frac{1}{p}T(p)=\frac{s}{u}T(\frac{u}{s})\;\;where\;\;\frac{s}{u}=\frac{1}{p}. & \end{array}$$ (29)

Now, Elzaki transform is define by $T(p)=E\{f(t)\}=p\int_{0}^{\infty }e^{\frac{-t}{p}}f(t)dt,$ by substituting, $p=\frac{u}{s}$ then, we have


$$\begin{array}{ccc} T(p) & =E\{f(t)\}=p\int_{0}^{\infty }e^{\frac{-t}{p}}f(t)dt=\frac{u}{s}\int_{0}^{\infty }e^{\frac{-st}{u}}f(t)dt=\frac{u}{s}\hat{V}(s,u), & \end{array}$$ (30)

$$\begin{array}{cc} T(\frac{u}{s}) & =\frac{u}{s}\hat{V}(s,u)\;\;where\;\;\frac{u}{s}=p. \end{array}$$ (31)

□

** Example 6. **Let us consider function $f(t)=e^{5t}$ the Elzaki transform $T(\frac{u}{s})$ and Shehu transform $\hat{V}(s,u)$ of the function, *f*(*t*) are given by, $T(\frac{u}{s})=E(e^{5t})=\frac{(\frac{u}{s}{)}^{2}}{1-5(\frac{u}{s})}=\frac{(\frac{u^{2}}{s})}{s-5u}=\frac{u}{s}(\frac{u}{s-5u}),\;\;but\;\;\hat{V}(s,u)=S\{e^{5t}\}=\frac{u}{s-5u},$ therefore, we obtain the required result, $T(\frac{u}{s})=\frac{u}{s}(\frac{u}{s-5u})=\frac{u}{s}\hat{V}(s,u)\;\;where\;\;\frac{u}{s}=p.$

**Duality between Shehu and Kamal Transform**

Let *Ĝ* ( *p* ) ,  is Kamal transform of the function *f*(*t*) define by, $Ĝ(p)=K\{f(t)\}=\int_{0}^{\infty }e^{\frac{-t}{p}}f(t)dt.$

** Theorem 3.7. ** Let the function *f* ( *t* ) ∈ *V* has Shehu transform $S\{f(t)\}=\hat{V}(s,u)$ and Kamal transform *Ĝ* ( *p* ) = *K* { *f* ( *t* ) } ,  then $\hat{V}(s,u)=Ĝ(\frac{u}{s}).$

** Proof. **Since Shehu transform $\hat{V}(s,u)$ of the function *f*(*t*) is define as, $\hat{V}(s,u)=S(f(t))=\int_{0}^{\infty }e^{\frac{-st}{u}}f(t)dt,$ by substituting, $\frac{s}{u}=\frac{1}{p}$ then, we have


$$\begin{array}{ccc} \hat{V}(s,u) & =S\{f(t)\}=\int_{0}^{\infty }e^{\frac{-st}{u}}f(t)dt=\int_{0}^{\infty }e^{\frac{-t}{p}}f(t)dt=\int_{0}^{\infty }e^{\frac{-t}{p}}f(t)dt=Ĝ(p) & \end{array}$$ (32)

$$\begin{array}{ccc} \hat{V}(s,u) & =Ĝ(\frac{u}{s})\;\;where\;\;p=\frac{u}{s}. & \end{array}$$ (33)

Now, Kamal transform is define by, $Ĝ(p)=K\{f(t)\}=\int_{0}^{\infty }e^{\frac{-t}{p}}f(t)dt,$ by substituting, $p=\frac{u}{s}$ then, we have


$$\begin{array}{ccc} Ĝ(p) & = & \int_{0}^{\infty }e^{\frac{-t}{p}}f(t)dt=\int_{0}^{\infty }e^{\frac{-st}{u}}f(t)dt=\hat{V}(s,u), \end{array}$$ (34)

$$\begin{array}{ccc} Ĝ(\frac{u}{s}) & = & \hat{V}(s,u)\;\;where\;\;p=\frac{u}{s}. \end{array}$$ (35)

□

** Example 7. **Let function $f(t)=e^{3t},$ the Kamal transform $Ĝ(p)=Ĝ(\frac{u}{s})$ and Shehu transform $\hat{V}(s,u)$ of the function *f*(*t*) are given by, $\hat{V}(s,u)=S\{e^{3t}\}=\frac{u}{s-3u}.$ Now, take Kamal transform of the function *f*(*t*) as


$$\begin{array}{cc} Ĝ(\frac{u}{s})=K(e^{3t})=\frac{\frac{u}{s}}{1-3(\frac{u}{s})}=\frac{u}{s-3u}=\hat{V}(s,u). & \end{array}$$

**Duality between Shehu and Fourier Transform**

Let *X* ( *w* ) ,  is Fourier transform of a function *f*(*t*) define by, $X(w)=X\{f(t)\}=\int_{-\infty }^{\infty }e^{-iwt}f(t)dt.$

** Theorem 3.8. ** Let *f* ( *t* ) ∈ *V* and $S\{f(t)\}=\hat{V}(s,u)$ and *X* ( *w* ) = *X* { *f* ( *t* ) } ,  then $\hat{V}(s,u)=X(\frac{s}{iu})$ and $\hat{V}(i,\frac{1}{w})=X(w).$

** Proof. **Since Shehu transform is define as $\hat{V}(s,u)=S(f(t))=\int_{0}^{\infty }e^{\frac{-st}{u}}f(t)dt,$ by substituting, $\frac{s}{u}=iw,$ we have


$$\begin{array}{ccc} \hat{V}(s,u)=\int_{0}^{\infty }e^{\frac{-st}{u}}f(t)dt=\int_{0}^{\infty }e^{-iwt}f(t)dt=X(w)=X(\frac{s}{iu}),\;\;where\;\;t\in [0,\infty ). & & \end{array}$$ (36)

Fourier transform of *f*(*t*) is define as $X(w)=X\{f(t)\}=\int_{-\infty }^{\infty }e^{-iwt}f(t)dt,$ by substituting, $iw=\frac{s}{u},$ then we have


$$X(\frac{s}{iu})=\int_{-\infty }^{\infty }e^{\frac{-s}{u}t}f(t)dt=\hat{V}(s,u).$$

□

** Example 8. **Let $f(t)=e^{-5t},$ then Fourier and Shehu transform are given by,


$$\begin{array}{cc} X(w)=F(e^{-5t})=\frac{1}{5+sw},\;\;and\;\;\hat{V}(s,u)=\hat{V}(s,\frac{1}{w})=S\{e^{-5t}\}=\frac{1}{sw+5}. & \end{array}$$

**Duality between Shehu and Aboodh Transform**

Let $A(p^{\prime }),$ is Aboodh transform of the function *f*(*t*) define by $A(p^{\prime })=Â\{f(t)\}=\frac{1}{p}\int_{0}^{\infty }e^{-pt}f(t)dt.$

** Theorem 3.9. ** Let *f* ( *t* ) ∈ *V* and $S\{f(t)\}=\hat{V}(s,u)$ and *A* ( *s* ) = *Â* { *f* ( *t* ) } ,  then $\hat{V}(s,u)=\frac{s}{u}Â(\frac{s}{u}),$ and $A(\frac{s}{u})=\frac{u}{s}\hat{V}(s,u).$

** Proof. **Since Shehu transform is define as, $\hat{V}(s,u)=S(f(t))=\int_{0}^{\infty }e^{\frac{-s}{u}t}f(t)dt,$ by substituting $\frac{s}{u}=p^{\prime }$ then, we have


$$\begin{array}{cccc} \hat{V}(s,u) & =\int_{0}^{\infty }e^{\frac{-s}{u}t}f(t)dt=\frac{p^{\prime }}{p^{\prime }}\int_{0}^{\infty }e^{-p^{\prime }t}f(t)dt=p^{\prime }A(p^{\prime }), & & \\ \hat{V}(s,u) & =\frac{s}{u}A(\frac{s}{u})\;\;where\;\;\frac{s}{u}=p^{\prime }. & \end{array}$$ (37)

Using the definition of Aboodh transform $A(p^{\prime })=Â\{f(t)\}=\frac{1}{p^{\prime }}\int_{0}^{\infty }e^{-p^{\prime }t}f(t)dt$ by substituting, $p^{\prime }=\frac{s}{u}$ then, we have


$$\begin{array}{ccc} A(p^{\prime }) & = & \frac{1}{p^{\prime }}\int_{0}^{\infty }e^{-p^{\prime }t}f(t)dt=\frac{u}{s}\int_{0}^{\infty }e^{\frac{-s}{u}t}f(t)dt\Rightarrow A(\frac{s}{u})=\frac{u}{s}\hat{V}(s,u). \end{array}$$ (38)

□

** Example 9. **Let us consider the function $f(t)=e^{4t},$ the Shehu transform of $\hat{V}(s,u)$ of the function *f*(*t*) is given by $\hat{V}(s,u)=S\{e^{4t}\}=\frac{u}{s-4u}.$ Now, the Aboodh transform $A(\frac{s}{u})$ of the function *f*(*t*) provide us


$$\begin{array}{cc} A(\frac{s}{u})=Â(e^{4t})=\frac{1}{{\left(\frac{s}{u}\right)}^{2}-4(\frac{s}{u})}=\frac{u}{s}(\frac{u}{s-4u})=\frac{u}{s}\hat{V}(s,u). & \end{array}$$

** Remark 1. ** ∙  From Eq. (12) and Eq. (15) we can easily obtain the duality relation between the Laplace and natural transforms as follows $F(\frac{s}{u})=uR(\frac{s}{u},u).$  ∙  Similarly, Eq. (12) and Eq. (18) provide us the duality relation between the natural and Sumudu transforms as follows $R(s,u)=\frac{1}{s}G(\frac{u}{s}).$  ∙  From Eq. (12) and Eq. (36) the duality relation between the natural and Fourier transforms is obtained as follows $R(s,u)=\frac{1}{u}X(\frac{s}{iu}).$  ∙  The duality relation between the Mellin and natural transforms is obtained from Eq. (12) and Eq. (23) as follows R(s,u)=1uM{f(−ln ⁡ t),su}. These are the duality relations discussed in the paper [29].

** Remark 2. ** ∙  From Eq. (15) and Eq. (18) we can easily obtain the duality relation between the Laplace and Sumudu transforms as follows $F(\frac{s}{u})=\frac{u}{s}G(\frac{u}{s}).$  ∙  Similarly, the duality between the Fourier and Sumudu transforms is obtained from Eq. (18) and Eq. (36) as follows $X(\frac{s}{iu})=\frac{u}{s}G(\frac{u}{s}).$ **Table 1** Shows the list of the Shehu, natural, Laplace, Sumudu, Laplace-Carson, Kamal, Elzaki and Aboodh transforms of the basic functions from the dualities relation with Shehu transform.  ∙  Moreover, the duality relation between the Mellin and Sumudu transform is obtained from Eq. (18) and Eq. (23) as G(us)=usM{f(−ln ⁡ t),su}. These are the duality relations discussed in the paper [28].

**Table 1:** List of the Shehu, natural, Laplace, Sumudu, Laplace-Carson, Kamal, Elzaki and Aboodh transforms of the basic functions from the dualities relation with Shehu transform

S. No	f(t)	*S* { *f* ( *t* ) } $=\hat{V}\text{(s,u)}$	R(s,u) = $\frac{1}{u}\hat{V}(s,u)$	F(s) = $\hat{V}(us,u)$	*G*(*p*) $= G(\frac{u}{s})$ $=\frac{s}{u}\hat{V}\text{(s,u)}$	${\hat{f}}_{c}(\frac{1}{p})$ $={\hat{f}}_{c}(\frac{s}{u})$ $=\frac{s}{u}\hat{V}(s,u)$	*Ĝ* ( *p* ) $= Ĝ(\frac{u}{s})$ $=\hat{V}(s,u)$	*T*(*p*) $= T(\frac{u}{s})$ $=\frac{u}{s}\hat{V}(s,u)$	$A(\frac{1}{p})$ $= A(\frac{s}{u})$ $=\frac{u}{s}\hat{V}(s,u)$
1	1	$\frac{u}{s}$	$\frac{1}{s}$	$\frac{1}{s}$	1	1	$\frac{u}{s}=p$	$\frac{u^{2}}{s^{2}}=p^{2}$	$\frac{u^{2}}{s^{2}}=p^{2}$
2	t	$\frac{u^{2}}{s^{2}}$	$\frac{u}{s^{2}}$	$\frac{1}{s^{2}}$	$\frac{u}{s}=p$	$\frac{u}{s}=p$	$\frac{u^{2}}{s^{2}}=p^{2}$	$\frac{u^{3}}{s^{3}}=p^{3}$	$\frac{u^{3}}{s^{3}}=p^{3}$
3	$\frac{t^{n}}{n!}$	$\frac{u^{n+1}}{s^{n+1}}$	$\frac{u^{n}}{s^{n+1}}$	$\frac{1}{s^{n+1}}$	${\left(\frac{u}{s}\right)}^{n}$	${\left(\frac{u}{s}\right)}^{n}$	${\left(\frac{u}{s}\right)}^{n+1}$	${\left(\frac{u}{s}\right)}^{n+2}$	${\left(\frac{u}{s}\right)}^{n+2}$
	*n* ∈ *N*				$=p^{n}$	$=p^{n}$	$=p^{n+1}$	$=p^{n+2}$	$=p^{n+2}$
4	$\frac{t^{n}}{\Gamma (n+1)}$	$\frac{u^{n+1}}{s^{n+1}}$	$\frac{u^{n}}{s^{n+1}}$	$\frac{1}{s^{n+1}}$	${\left(\frac{u}{s}\right)}^{n}$	${\left(\frac{u}{s}\right)}^{n}$	${\left(\frac{u}{s}\right)}^{n+1}$	${\left(\frac{u}{s}\right)}^{n+2}$	${\left(\frac{u}{s}\right)}^{n+2}$
	*n* ∈ *N*				$=p^{n}$	$=p^{n}$	$=p^{n+1}$	$=p^{n+2}$	$=p^{n+2}$
5	*sin* ⁡ ( *t* )	$\frac{u^{2}}{s^{2}+u^{2}}$	$\frac{u}{s^{2}+u^{2}}$	$\frac{1}{s^{2}+}$	$\frac{us}{s^{2}+u^{2}}$	$\frac{us}{s^{2}+u^{2}}$	$\frac{u^{2}}{s^{2}+u^{2}}$	$\frac{u^{3}}{s^{3}+su^{2}}$	$\frac{u^{3}}{s^{3}+su^{2}}$
					$=\frac{p}{1+p^{2}}$	$=\frac{p}{1+p^{2}}$	$=\frac{p^{2}}{1+p^{2}}$	$=\frac{p^{3}}{1+p^{2}}$	$=\frac{p^{3}}{1+p^{2}}$
6	*sinh* ⁡ ( *t* )	$\frac{u^{2}}{s^{2}-u^{2}}$	$\frac{u}{s^{2}-u^{2}}$	$\frac{1}{s^{2}-1}$	$\frac{us}{s^{2}-u^{2}}$	$\frac{us}{s^{2}-u^{2}}$	$\frac{u^{2}}{s^{2}-u^{2}}$	$\frac{u^{3}}{s^{3}-su^{2}}$	$\frac{u^{3}}{s^{3}-su^{2}}$
					$=\frac{p}{1-p^{2}}$	$=\frac{p}{1-p^{2}}$	$=\frac{p^{2}}{1-p^{2}}$	$=\frac{p^{3}}{1-p^{2}}$	$=\frac{p^{3}}{1-p^{2}}$
7	*cos* ⁡ ( *t* )	$\frac{us}{s^{2}+u^{2}}$	$\frac{s}{s^{2}+u^{2}}$	$\frac{s}{s^{2}+1}$	$\frac{s^{2}}{s^{2}+u^{2}}$	$\frac{s^{2}}{s^{2}+u^{2}}$	$\frac{us}{s^{2}+u^{2}}$	$\frac{u^{2}}{s^{2}+u^{2}}$	$\frac{u^{2}}{s^{2}+u^{2}}$
					$=\frac{1}{1+p^{2}}$	$=\frac{1}{1+p^{2}}$	$=\frac{p}{1+p^{2}}$	$=\frac{p^{2}}{1+p^{2}}$	$=\frac{p^{2}}{1+p^{2}}$
8	*cosh* ⁡ ( *t* )	$\frac{us}{s^{2}-u^{2}}$	$\frac{s}{s^{2}-u^{2}}$	$\frac{s}{s^{2}-1}$	$\frac{s^{2}}{s^{2}-u^{2}}$	$\frac{s^{2}}{s^{2}-u^{2}}$	$\frac{us}{s^{2}-u^{2}}$	$\frac{u^{2}}{s^{2}-u^{2}}$	$\frac{u^{2}}{s^{2}-u^{2}}$
					$=\frac{1}{1-p^{2}}$	$=\frac{1}{1-p^{2}}$	$=\frac{p}{1-p^{2}}$	$=\frac{p^{2}}{1-p^{2}}$	$=\frac{p^{2}}{1-p^{2}}$
9	$e^{t}$	$\frac{u}{s-u}$	$\frac{1}{s-u}$	$\frac{1}{s-1}$	$\frac{s}{s-u}$	$\frac{s}{s-u}$	$\frac{u}{s-u}$	$\frac{u^{2}}{s^{2}-su}$	$\frac{u^{2}}{s^{2}-su}$
					$=\frac{1}{1-p}$	$=\frac{1}{1-p}$	$=\frac{p}{1-p}$	$=\frac{p^{2}}{1-p}$	$=\frac{p^{2}}{1-p}$
10	$\frac{\sin(at)}{a}$	$\frac{u^{2}}{s^{2}+a^{2}u^{2}}$	$\frac{u}{s^{2}+a^{2}u^{2}}$	$\frac{1}{s^{2}+a^{2}}$	$\frac{us}{s^{2}+a^{2}u^{2}}$	$\frac{us}{s^{2}+a^{2}u^{2}}$	$\frac{u^{2}}{s^{2}+a^{2}u^{2}}$	$\frac{u^{3}}{s^{3}+a^{2}su^{2}}$	$\frac{u^{3}}{s^{3}+a^{2}su^{2}}$
					$=\frac{p}{1+a^{2}p^{2}}$	$=\frac{p}{1+a^{2}p^{2}}$	$=\frac{p^{2}}{1+a^{2}p^{2}}$	$=\frac{p^{3}}{1+a^{2}p^{2}}$	$=\frac{p^{3}}{1+a^{2}p^{2}}$
11	$\frac{\sinh(at)}{a}$	$\frac{u^{2}}{s^{2}-a^{2}u^{2}}$	$\frac{u}{s^{2}-a^{2}u^{2}}$	$\frac{a}{s^{2}-a^{2}}$	$\frac{us}{s^{2}-a^{2}u^{2}}$	$\frac{us}{s^{2}-a^{2}u^{2}}$	$\frac{u^{2}}{s^{2}-a^{2}u^{2}}$	$\frac{u^{3}}{s^{3}-a^{2}su^{2}}$	$\frac{u^{3}}{s^{3}-a^{2}su^{2}}$
					$=\frac{p}{1-a^{2}p^{2}}$	$=\frac{p}{1-a^{2}p^{2}}$	$=\frac{p^{2}}{1-a^{2}p^{2}}$	$=\frac{p^{3}}{1-a^{2}p^{2}}$	$=\frac{p^{3}}{1-a^{2}p^{2}}$
12	*cos* ⁡ ( *at* )	$\frac{us}{s^{2}+a^{2}u^{2}}$	$\frac{s}{s^{2}+a^{2}u^{2}}$	$\frac{s}{s^{2}+a^{2}}$	$\frac{s^{2}}{s^{2}+a^{2}u^{2}}$	$\frac{s^{2}}{s^{2}+a^{2}u^{2}}$	$\frac{us}{s^{2}+a^{2}u^{2}}$	$\frac{u^{2}}{s^{2}+a^{2}u^{2}}$	$\frac{u^{2}}{s^{2}+a^{2}u^{2}}$
					$=\frac{1}{1+a^{2}p^{2}}$	$=\frac{1}{1+a^{2}p^{2}}$	$=\frac{p}{1+a^{2}p^{2}}$	$=\frac{p^{2}}{1+a^{2}p^{2}}$	$=\frac{p^{2}}{1+a^{2}p^{2}}$
13	*cosh* ⁡ ( *at* )	$\frac{us}{s^{2}-a^{2}u^{2}}$	$\frac{s}{s^{2}-a^{2}u^{2}}$	$\frac{s}{s^{2}-a^{2}}$	$\frac{s^{2}}{s^{2}-a^{2}u^{2}}$	$\frac{s^{2}}{s^{2}-a^{2}u^{2}}$	$\frac{us}{s^{2}-a^{2}u^{2}}$	$\frac{u^{2}}{s^{2}-a^{2}u^{2}}$	$\frac{u^{2}}{s^{2}-a^{2}u^{2}}$
					$=\frac{1}{1-a^{2}p^{2}}$	$=\frac{1}{1-a^{2}p^{2}}$	$=\frac{p}{1-a^{2}p^{2}}$	$=\frac{p^{2}}{1-a^{2}p^{2}}$	$=\frac{p^{2}}{1-a^{2}p^{2}}$
14	$e^{at}$	$\frac{u}{s-au}$	$\frac{1}{s-au}$	$\frac{1}{s-a}$	$\frac{s}{s-au}$	$\frac{s}{s-au}$	$\frac{u}{s-au}$	$\frac{u^{2}}{s^{2}-asu}$	$\frac{u^{2}}{s^{2}-asu}$
					$=\frac{1}{1-ap}$	$=\frac{1}{1-ap}$	$=\frac{p}{1-ap}$	$=\frac{p^{2}}{1-ap}$	$=\frac{p^{2}}{1-ap}$

** Remark 3. ** ∙  From Eq. (15) and Eq. (26) we can easily obtain the duality relation between the Laplace and Laplace-Carson transforms as follows $F(s)=\frac{1}{s}{\hat{f}}_{c}(s).$  ∙  Also from Eq. (18) and Eq. (26) we can easily obtain the duality relation between the Laplace-Carson and Sumudu transforms as follows $G(\frac{u}{s})={\hat{f}}_{c}(\frac{s}{u}).$  ∙  Also from Eq. (12) and Eq. (26) we can easily obtain the duality relation between the natural and Laplace-Carson transforms as follows $R(s,u)=\frac{1}{s}{\hat{f}}_{c}(\frac{s}{u}).$  ∙  The duality relation between the Laplace-Carson and Kamal transforms is obtained from Eq. (26) and Eq. (33) as $Ĝ(\frac{u}{s})=\frac{u}{s}{\hat{f}}_{c}(\frac{s}{u}).$  ∙  The Laplace-Carson and Aboodh transforms duality is achieved from the Eq. (26) and Eq. (37), $A(\frac{s}{u})=(\frac{u}{s}{)}^{2}{\hat{f}}_{c}(\frac{s}{u}).$  ∙  Moreover, the duality relation between the Laplace-Carson and Elzaki transforms is obtained from Eq. (26) and Eq. (29) as $\frac{s}{u}T(\frac{u}{s})=(\frac{u}{s}{)}^{2}{\hat{f}}_{c}(\frac{s}{u}).$ These are the duality relations discussed in the paper [30].

** Remark 4. **Duality relations of all the integral transforms with one another can be easily obtained by comparing any two transforms with Shehu transform, we discussed the above only for samples, therefore Shehu transform is used as a source to connect the integral transforms.

## 4 Application of the duality relation to fractional order differential equations

** Theorem 4.1. ** Let *y* ( *t* ) ∈ *V* ,  the Shehu transform of *y*(*t*) is given by $S\{y(t)\}=\hat{V}(s,u)$ and Laplace transform is $\{Ly(t)=F(\frac{s}{u})\},$ then $S{(}^{c}D_{0}^{\alpha }y(t))=L{(}^{c}D_{0}^{\alpha }y(t)).$

** Proof. **We know from theorem 2.10 that Shehu transform of caputo fractional order derivative is given by


$$S{(}^{c}D_{0}^{\alpha }y(t))={\left(\frac{s}{u}\right)}^{\alpha }\hat{V}(s,u)-\overset{n-1}{\underset{k=0}{\sum }}{\left(\frac{s}{u}\right)}^{\alpha -(k+1)}y^{k}(0),where,\;n-1\leq \alpha <n$$

Let $s=us^{\prime }$ then $s^{\prime }=\frac{s}{u}$ we have


$$\begin{array}{llll} S{(}^{c}D_{0}^{\alpha }y(t)) & ={\left(s^{\prime }\right)}^{\alpha }\hat{V}(us^{\prime },u)-\overset{n-1}{\underset{k=0}{\sum }}{\left(s^{\prime }\right)}^{\alpha -(k+1)}y^{k}(0),usingtheorem\;3.2\; & & \;\\ & ={\left(s^{\prime }\right)}^{\alpha }F(s^{\prime })-\overset{n-1}{\underset{k=0}{\sum }}{\left(s^{\prime }\right)}^{\alpha -(k+1)}y^{k}(0)={\left(\frac{s}{u}\right)}^{\alpha }F(\frac{s}{u})-\overset{n-1}{\underset{k=0}{\sum }}{\left(\frac{s}{u}\right)}^{\alpha -(k+1)}y^{k}(0),\; & & \;\\ & \;usingde\mathrm{fi}nition\;2.11\; & & \;\\ & =L{(}^{c}D_{0}^{\alpha }y(t)).\; & & \; \end{array}$$

□

** Example 10. **Let us consider the fractional differential equation


$$\begin{array}{ccc} \{\begin{array}{ccc} {\;}^{c}D_{0}^{2}y(t){+}^{c}D_{0}^{\frac{3}{2}}y(t)+y(t) & = & t+1\\ y(0)=y^{\prime }(0)=1.\; \end{array} & & \end{array}$$ (39)

Taking the Shehu transform of Eq. (39) as follows


$$\begin{array}{rcll} \left({\left(\frac{s}{u}\right)}^{2}+{\left(\frac{s}{u}\right)}^{\frac{3}{2}}+1)\hat{V}(s,u\right) & = & \frac{s}{u}+{\left(\frac{s}{u}\right)}^{\frac{1}{2}}+{\left(\frac{s}{u}\right)}^{-\frac{1}{2}}+1+\frac{u}{s}+{\left(\frac{u}{s}\right)}^{2} & \\ \hat{V}(s,u) & = & \frac{\frac{s}{u}+(\frac{s}{u}{)}^{\frac{1}{2}}+\frac{u}{s}}{(\frac{s}{u}{)}^{2}+(\frac{s}{u}{)}^{\frac{3}{2}}+1}+\frac{(\frac{u}{s}{)}^{2}+(\frac{u}{s}{)}^{\frac{1}{2}}+1}{(\frac{s}{u}{)}^{2}+(\frac{s}{u}{)}^{\frac{3}{2}}+1}=\frac{u}{s}+(\frac{u}{s}{)}^{2}. & \end{array}$$

The Laplace transform of equation (39) is given as


$$\begin{array}{rcll} \;{\left(s^{\prime }\right)}^{2}F(s^{\prime })-sy(0)-y^{\prime }(0)+\frac{{\left(s^{\prime }\right)}^{2}F(s^{\prime })-sy(0)-y^{\prime }(0)}{{s^{\prime }}^{\frac{1}{2}}}+F(s^{\prime }) & = & \frac{1}{s^{\prime }}+(\frac{1}{{s^{\prime }}^{2}}) & \\ ({\left(s^{\prime }\right)}^{2}+{s^{\prime }}^{\frac{3}{2}}+1)F(s^{\prime })=(\frac{1}{s^{\prime }}+(\frac{1}{{s^{\prime }}^{2}}))({\left(s^{\prime }\right)}^{2}+{s^{\prime }}^{\frac{3}{2}}+1)\Rightarrow F(s^{\prime }) & = & \frac{1}{s^{\prime }}+(\frac{1}{{s^{\prime }}^{2}}).\; & \end{array}$$

Now, replace $s^{\prime }=\frac{s}{u}$ then $F(\frac{s}{u})=\frac{s}{u}+{\left(\frac{s}{u}\right)}^{2}$ therefore, $F(\frac{s}{u})=\hat{V}(s,u).$ By taking the inverse Shehu and Laplace transform respectively, one can get the solution of equation (39) *y* ( *t* ) = *t* + 1 . 

** Theorem 4.2. ** Let *y* ( *t* ) ∈ *V* ,  the Shehu transform of *y*(*t*) is given by $S\{y(t)\}=\hat{V}(s,u)$ and Sumudu transform is $\hat{S}\{y(t)\}=G(\frac{u}{s}),$ then $S{(}^{c}D_{0}^{\alpha }y(t))=\frac{u}{s}\hat{S}{(}^{c}D_{0}^{\alpha }y(t)).$

** Proof. **We know from theorem 2.10 that Shehu transform of caputo fractional order derivative is given by


$$\begin{array}{llll} S{(}^{c}D_{0}^{\alpha }y(t)) & ={\left(\frac{s}{u}\right)}^{\alpha }\hat{V}(s,u)-\overset{n-1}{\underset{k=0}{\sum }}{\left(\frac{s}{u}\right)}^{\alpha -(k+1)}y^{k}(0),where,\;n-1\leq \alpha <n\; & & \;\\ & ={\left(\frac{s}{u}\right)}^{\alpha }\hat{V}(s,u)-\overset{n-1}{\underset{k=0}{\sum }}{\left(\frac{s}{u}\right)}^{\alpha -(k+1)}y^{k}(0)=\frac{u}{s}{\left(\frac{s}{u}\right)}^{\alpha }\frac{s}{u}\hat{V}(s,u)-\overset{n-1}{\underset{k=0}{\sum }}{\left(\frac{s}{u}\right)}^{\alpha -(k+1)}y^{k}(0),\; & & \;\\ & \;usingtheorem3.3 & & \;\\ & =\frac{u}{s}\{{\left(\frac{u}{s}\right)}^{-\alpha }G(\frac{u}{s})-\overset{n-1}{\underset{k=0}{\sum }}{\left(\frac{u}{s}\right)}^{-\alpha +k}y^{k}(0)\}=\frac{u}{s}\{{\left(\frac{u}{s}\right)}^{-\alpha }\{G(\frac{u}{s})-\overset{n-1}{\underset{k=0}{\sum }}{\left(\frac{u}{s}\right)}^{k}y^{k}(0)\}\},\; & & \;\\ & \;usingtheorem2.12 & & \;\\ & =\frac{u}{s}\hat{S}{(}^{c}D_{0}^{\alpha }y(t)).\; & & \; \end{array}$$

□

** Example 11. ** Let us consider the fractional differential equation


$$\begin{array}{ccc} \{\begin{array}{ccc} {\;}^{c}D_{0}^{\frac{1}{2}}y(t)+y(t) & = & t^{2}+\frac{\Gamma (3)}{\Gamma (\frac{5}{2})}t^{2},where,\;t>0,\\ y(0)=0.\;\;\;\;\; \end{array} & & \end{array}$$ (40)

Taking the Shehu transform of Eq. (40) as follows


$$\begin{array}{rcll} \left({\left(\frac{s}{u}\right)}^{\frac{1}{2}}+1)\hat{V}(s,u\right) & = & 2{\left(\frac{u}{s}\right)}^{3}+\Gamma (3){\left(\frac{u}{s}\right)}^{\frac{5}{2}}, & \end{array}$$

$$\begin{array}{ccc} \hat{V}(s,u)=\frac{2{\left(\frac{u}{s}\right)}^{3}+\Gamma (3){\left(\frac{u}{s}\right)}^{\frac{5}{2}}}{{\left(\frac{s}{u}\right)}^{\frac{1}{2}}+1}=\frac{2{\left(\frac{u}{s}\right)}^{3}(1+{\left(\frac{s}{u}\right)}^{\frac{1}{2}})}{{\left(\frac{s}{u}\right)}^{\frac{1}{2}}+1}=2(\frac{u}{s}{)}^{3}. & & \end{array}$$ (41)

Now, the sumudu transform of equation (40) is given as


$$\begin{array}{rcll} \frac{G(u^{\prime })}{{u^{\prime }}^{\frac{1}{2}}}+G(u^{\prime })=2{u^{\prime }}^{2}+2{u^{\prime }}^{\frac{3}{2}}\Rightarrow (\frac{{u^{\prime }}^{\frac{1}{2}}+1}{{u^{\prime }}^{\frac{1}{2}}})G(u^{\prime }) & = & 2{u^{\prime }}^{2}+2{u^{\prime }}^{\frac{3}{2}} & \\ G(u^{\prime })=\frac{2{u^{\prime }}^{\frac{5}{2}}}{{u^{\prime }}^{\frac{1}{2}}+1}+\frac{2{u^{\prime }}^{2}}{{u^{\prime }}^{\frac{1}{2}}+1}=\frac{2{u^{\prime }}^{2}({u^{\prime }}^{\frac{1}{2}})+1}{{u^{\prime }}^{\frac{1}{2}}+1} & = & 2{u^{\prime }}^{2}. & \end{array}$$

Replace, $u^{\prime }$ with $\frac{u}{s}$, one can get.


$$\begin{array}{ccc} G(\frac{u}{s})=(\frac{u}{s}{)}^{2}\;\;\;thisimplies\;\;\frac{u}{s}G(\frac{u}{s})=(\frac{u}{s}{)}^{3}=\hat{V}(s,u). & & \end{array}$$ (42)

$$\begin{array}{rcll} Now,\;\;\frac{u}{s}G(\frac{u}{s}) & = & (\frac{u}{s}{)}^{3}=\hat{V}(s,u). & \end{array}$$

By taking the inverse Shehu and Laplace transform of equations 41 and 42 respectively, one can get the solution of equation(40), $y(t)=t^{2}.$

** Theorem 4.3. ** Let *y* ( *t* ) ∈ *V* ,  the Shehu transform of *y*(*t*) is given by $S\{y(t)\}=\hat{V}(s,u)$ and natural transform is *N* { *y* ( *t* ) } = *R* ( *s* , *u* ) ,  then $S{(}^{c}D_{0}^{\alpha }y(t))=uN{(}^{c}D_{0}^{\alpha }y(t)).$

** Proof. **We know from theorem 2.10 that Shehu transform of caputo fractional order derivative is given by


$$\begin{array}{llll} S{(}^{c}D_{0}^{\alpha }y(t)) & ={\left(\frac{s}{u}\right)}^{\alpha }\hat{V}(s,u)-\overset{n-1}{\underset{k=0}{\sum }}{\left(\frac{s}{u}\right)}^{\alpha -(k+1)}y^{k}(0),where,\;n-1\leq \alpha <n\; & & \;\\ S{(}^{c}D_{0}^{\alpha }y(t)) & ={\left(\frac{s}{u}\right)}^{\alpha }\hat{V}(s,u)-\overset{n-1}{\underset{k=0}{\sum }}{\left(\frac{s}{u}\right)}^{\alpha -(k+1)}y^{k}(0),substitute,\;s=us^{\prime }\; & & \;\\ & ={s^{\prime }}^{\alpha }\hat{V}(us^{\prime },u)-\overset{n-1}{\underset{k=0}{\sum }}{\left(s^{\prime }\right)}^{\alpha -(k+1)}y^{k}(0)\; & & \;\\ & =u{s^{\prime }}^{\alpha }\frac{1}{u}\hat{V}(us^{\prime },u)-\overset{n-1}{\underset{k=0}{\sum }}{\left(s^{\prime }\right)}^{\alpha -(k+1)}y^{k}(0),usingtheorem3.1 & & \;\\ & =u{s^{\prime }}^{\alpha }R(s^{\prime },u)-\overset{n-1}{\underset{k=0}{\sum }}{\left(s^{\prime }\right)}^{\alpha -(k+1)}y^{k}(0),now,replace\;s^{\prime }\;with\;\frac{s}{u}\; & & \;\\ & =u\{{\left(\frac{s}{u}\right)}^{\alpha }R(\frac{s}{u},u)-\overset{n-1}{\underset{k=0}{\sum }}\frac{s^{\alpha -(k+1)}}{u^{\alpha -k}}y^{k}(0)\},usingproposition2.1 & & \;\\ & =uN{(}^{c}D_{0}^{\alpha }y(t)).\; & & \; \end{array}$$

□

** Example 12. **Let us again consider the fractional differential equation (40).


$$\begin{array}{ccc} \{\begin{array}{ccc} {\;}^{c}D_{0}^{\frac{1}{2}}y(t)+y(t) & =t^{2}+\frac{\Gamma (3)}{\Gamma (\frac{5}{2})}t^{2},where,\;t>0, & \\ y(0)=0.\;\;\;\;\; \end{array} & & \end{array}$$ (43)

From example 11 the Shehu transform of Eq. (41) is given as


$$\begin{array}{ccc} \hat{V}(s,u)=\frac{2{\left(\frac{u}{s}\right)}^{3}+\Gamma (3){\left(\frac{u}{s}\right)}^{\frac{5}{2}}}{{\left(\frac{s}{u}\right)}^{\frac{1}{2}}+1}=\frac{2{\left(\frac{u}{s}\right)}^{3}(1+(\frac{s}{u}{)}^{\frac{1}{2}})}{{\left(\frac{s}{u}\right)}^{\frac{1}{2}}+1}=2(\frac{u}{s}{)}^{3}. & & \end{array}$$ (44)

The natural transform of equation(40) is obtain as


$$\begin{array}{rcll} (\frac{s}{u}{)}^{\frac{1}{2}}R(s,u)+R(s,u)=2(\frac{u^{2}}{s^{3}})+2(\frac{u^{\frac{3}{2}}}{s^{\frac{5}{2}}})\Rightarrow ({\left(\frac{s}{u}\right)}^{\frac{1}{2}}+1)R(s,u) & = & 2(\frac{u^{2}}{s^{3}})(1+(\frac{s}{u}{)}^{\frac{1}{2}}). & \end{array}$$

$$\begin{array}{ccc} R(s,u)=2(\frac{u^{2}}{s^{3}}). & & \end{array}$$ (45)

$$FromEq.(44)andEq.(45),weobtainthedaulityrelation,\;\;uR(s,u)=\hat{V}(s,u).\;$$

By taking the inverse Shehu and Laplace transform of equations 44 and 45 respectively, one can get the solution of equation(40), $y(t)=t^{2}.$

** Example 13. ** Let us consider the non-linear fractional order differential equation ${\;}^{c}D_{0}^{\alpha }y(t)+a{\left(y(t)\right)}^{m}=g(t).$ If the Shehu transform of *y*(*t*) and *g*(*t*) are respectively $S\{y(t)\}=\hat{V}(s,u)$ and $S\{g(t)\}=V_{g}(s,u).$ Now, using theorem (2.10) and theorem (2.7) to find Shehu transform of the non-linear fractional differential equation


$${\;}^{c}D_{0}^{\alpha }y(t)+a{\left(y(t)\right)}^{m}=g(t)\;\;\Rightarrow S{(}^{c}D_{0}^{\alpha }y(t)+a{\left(y(t)\right)}^{m})=S\{g(t)\}$$

$$\begin{array}{cc} {\left(\frac{s}{u}\right)}^{\alpha }\hat{V}(s,u)-\overset{n-1}{\underset{k=0}{\sum }}{\left(\frac{s}{u}\right)}^{\alpha -(k+1)}y^{k}(0)+{\left(\frac{s}{u}\right)}^{m}\hat{V}(s,u)-\overset{m-1}{\underset{k=0}{\sum }}{\left(\frac{s}{u}\right)}^{\alpha -(k+1)}y^{k}(0)=V_{g}(s,u)\; & \\ \;where,\;n-1\leq \alpha <n & \end{array}$$

$$\{(\frac{s}{u}{)}^{\alpha }+(\frac{s}{u}{)}^{m}\}\hat{V}(s,u)=V_{g}(s,u)+\overset{n-1}{\underset{k=0}{\sum }}{\left(\frac{s}{u}\right)}^{\alpha -(k+1)}y^{k}(0)+\overset{m-1}{\underset{k=0}{\sum }}{\left(\frac{s}{u}\right)}^{\alpha -(k+1)}y^{k}(0)$$

$$\begin{array}{ccc} \hat{V}(s,u)=\frac{V_{g}(s,u)+\overset{n-1}{\underset{k=0}{\sum }}{\left(\frac{s}{u}\right)}^{\alpha -(k+1)}y^{k}(0)+\overset{m-1}{\underset{k=0}{\sum }}{\left(\frac{s}{u}\right)}^{\alpha -(k+1)}y^{k}(0)}{(\frac{s}{u}{)}^{\alpha }+(\frac{s}{u}{)}^{m}}. & & \end{array}$$ (46)

Now, if the natural transform of $y(t)\text{and}g(t)\text{are respectively}N\{y(t)\}=R(\frac{s}{u},u)\text{and}N\{g(t)\}=R_{g}(\frac{s}{u},u)$ then using theorem (2.8) and proposition (2.1) to find natural transform of non-linear fractional differential equation as follows


$${\;}^{c}D_{0}^{\alpha }y(t)+a{\left(y(t)\right)}^{m}=g(t)\;\;\Rightarrow N{(}^{c}D_{0}^{\alpha }y(t)+a{\left(y(t)\right)}^{m})=N\{g(t)\}$$

$$\begin{array}{cc} {\left(\frac{s^{\prime }}{u}\right)}^{\alpha }R(s^{\prime },u)-\overset{n-1}{\underset{k=0}{\sum }}\frac{{\left(s^{\prime }\right)}^{\alpha -(k+1)}}{u^{\alpha -k}}y^{k}(0)+{\left(\frac{s^{\prime }}{u}\right)}^{m}R(s^{\prime },u)-\overset{m-1}{\underset{k=0}{\sum }}\frac{{\left(s^{\prime }\right)}^{\alpha -(k+1)}}{u^{\alpha -k}}y^{k}(0)=R_{g}(s^{\prime },u) & \\ where,\;n-1\leq \alpha <n & \end{array}$$

$$\{(\frac{s^{\prime }}{u}{)}^{\alpha }+(\frac{s^{\prime }}{u}{)}^{m}\}R(s^{\prime },u)=R_{g}(s^{\prime },u)+\overset{n-1}{\underset{k=0}{\sum }}{\left(\frac{s^{\prime }}{u}\right)}^{\alpha -(k+1)}y^{k}(0)+\overset{m-1}{\underset{k=0}{\sum }}{\left(\frac{s^{\prime }}{u}\right)}^{\alpha -(k+1)}y^{k}(0)$$

$$R(s^{\prime },u)=\frac{R_{g}(s^{\prime },u)+\overset{n-1}{\underset{k=0}{\sum }}(\frac{{\left(s^{\prime }\right)}^{\alpha -(k+1)}}{u^{\alpha -k}})y^{k}(0)+\overset{m-1}{\underset{k=0}{\sum }}(\frac{{\left(s^{\prime }\right)}^{\alpha -(k+1)}}{u^{\alpha -k}})y^{k}(0)}{(\frac{s^{\prime }}{u}{)}^{\alpha }+(\frac{s^{\prime }}{u}{)}^{m}}.$$

Substitute $s^{\prime }=\frac{s}{u},$ we can get


$$\begin{array}{ccc} R(\frac{s}{u},u)=\frac{R_{g}(\frac{s}{u},u)+\overset{n-1}{\underset{k=0}{\sum }}(\frac{s^{\alpha -(k+1)}}{u^{\alpha -k}})y^{k}(0)+\overset{m-1}{\underset{k=0}{\sum }}(\frac{s^{\alpha -(k+1)}}{u^{\alpha -k}})y^{k}(0)}{(\frac{s}{u}{)}^{\alpha }+(\frac{s}{u}{)}^{m}}. & & \end{array}$$ (47)

Multiply Eq. 46 by *u* to get the duality relations between Shehu and natural transforms


$$\begin{array}{ccc} uR(\frac{s}{u},u)=\frac{uR_{g}(\frac{s}{u},u)+\overset{n-1}{\underset{k=0}{\sum }}(\frac{s}{u}{)}^{\alpha -(k+1)}y^{k}(0)+\overset{m-1}{\underset{k=0}{\sum }}(\frac{s}{u}{)}^{\alpha -(k+1)}y^{k}(0)}{(\frac{s}{u}{)}^{\alpha }+(\frac{s}{u}{)}^{m}}. & & \end{array}$$ (48)

By comparing Eq. (46) and Eq. (48) it is clear that the duality relations between Shehu and natural transforms is also applicable to non-linear fractional order differential equations. Also from theorem (3.1) the duality relations between Shehu and natural transforms is $uR(\frac{s}{u},u)=\hat{V}(s,u)\text{and}uR(\frac{s}{u},u)=V_{g}(s,u),$ therefore Eq. (48) produce Eq. (46).

## 5 Conclusion and future direction

Integral transforms are widely used in the research articles in the literature, due to their interesting applications in the solutions of problems of applied science and engineering. In many situations, researchers feel difficulties in applying a transform to solve differential or integral equations, therefore it is more convenient to derive dualities relations between integral transforms. Shehu transform has the properties to converge to the well-known integral transforms used in the literature only by changing the space parameters.

- In this manuscript, we derived duality relations of the Shehu transform with natural, Sumudu, Laplace, Laplace-Carson, Fourier, Elzaki, Kamal, Aboodh, and Mellin transforms.
- We proved successfully that the said transforms can be derived from the Shehu transform by simple substitutions.
- We concluded that these transforms are closely related to one another and the Shehu transform works as a source to inter-convert these transforms only by changing the space parameters.
- We show that the solutions obtained by the duality relations and by direct use of the integral transforms are the same.
- In this study, we discussed the applications of the duality relations in the solutions of linear and non-linear fractional order differential equations.
- Moreover, in this study, we derived the duality relations of papers [28–30] in the remarks from our results.
- Furthermore, we provide a list in which the transforms of basic functions are obtained from the duality relations of other integral transforms with Shehu transform.

This study is very helpful in unifying the integral transforms in the future. This study is also interested in the research in the future to solve that problems with duality relations whose direct solutions with particular integral transform was difficult. These duality relations are also interested in solving the fractional order differential equations like [36,37] in the future.

## References

[1] Spiegel MR. Theory and problems of Laplace transforms, Schaums outline series. New York: McGraw-Hill; 1965.

[2] Bochner S, Chandrasekharan K. Fourier transforms. Princeton, NJ, USA: Princeton University Press; 1949.

[3] Morse PM, Feshbach H. Methods of theoretical physics. New York: McGraw-Hill; 1953, pp. 484–5.

[4] Makarov AM. Application of the Laplace-Carson method of integral transformation to the theory of unsteady visco-plastic flows. J Eng Phys Thermophys. 1970;19:94–99.

[5] Watugala GK. Sumudu transform a new integral transform to solve differential equations and control engineering problems. Int J Math Educ Sci Technol 1993;24(1):35–43. doi: 10.1080/0020739930240105

[6] Khan ZH, Khan WA. N-transform properties and application. Nust J Eng Sci. 2008;1:127–33.

[7] Elzaki TM. The new integral transform Elzaki transform. Global J Pure Appl Math. 2011;7:57–64.

[8] Aboodh KS. The new integral transform Aboodh transform. Global J Pure Appl Math. 2013;1:35–43.

[9] Kamal A, Sedeeg H. The new integral transform Kamal transform. Adv Theor Appl Math. 2016;4:451–58.

[10] Maitama S, Zhao W. New integral transform: Shehu transform a generalization of Sumudu and Laplace transform for solving differential equations. Int J Appl. 2019;17(2):167–90.

[11] Watugala GK. Sumudu transform, a new integral transform to solve differential equations and control engineering problems. Math Eng Indus. 1998;6(4):319–29.

[12] Dattoli G, Martinelli MR, Ricci PE. On new families of integral transforms for the solution of partial differential equations. Integral Transforms Spec Funct. 2005;8:661–7. doi: 10.1080/10652460500105966

[13] Weerakoon S. Application of Sumudu transform to partial differential equations. Int J Math Educ Sci Technol. 1994;25:277–83.

[14] Bulut H, Baskonus HM, Belgacem FBM. The analytical solution of some fractional ordinary differential equations by the Sumudu transform method. Abstr Appl Anal. 2013;Article ID 203875:1–6.

[15] Beghami W, Maayah B, Bushnaq S, Arqub OA. The Laplace optimized decomposition method for solving systems of partial differential equations of fractional order. Int J Appl Comput Math. 2022;8(52). doi: 10.1007/s40819-022-01256-x

[16] Maayah B, Moussaoui A, Bushnaq S, Arqub OA. The multistep Laplace optimized decomposition method for solving fractional-order coronavirus disease model (COVID-19) via the Caputo fractional approach. Mathematica. 2022;55:963–77.

[17] Belgacem FBM, Silambarasan R. Maxwells equations solutions by means of the Natural transform. Math Eng Sci Aerosp. 2012;3:313–23.

[18] Silambarasn R, Belgacem FBM. Applications of natural transform to Maxwell’s equations. In: Progress in Electromagnetics Research Symposium Proceedings, Suzhou, China, September 12–16, 2011.

[19] Loonker D, Banerji PK. Applications of natural transform to differential equations. J Indian Acad Math. 2013;35:151–8.

[20] Loonker D, Banerji PK. Solution of Fractional ordinary differential equations by natural transform. Int J Math Eng Sci. 2013;2(12):1–7.

[21] Akinyemi L, Iyiola OS. Exact and approximate solutions of time-fractional models arising from physics via Shehu transform. Math Method Appl Sci. 2020;43(12):7442–64.

[22] Kapoor M. Shehu transform on time-fractional Schrödinger equations––an analytical approach. Int J Nonlinear Sci. 2022;24(5). doi: 10.1515/ijnsns-2021-0423

[23] Jena SR, Sahu I. A novel approach for numerical treatment of traveling wave solution of ion acoustic waves as a fractional nonlinear evolution equation on Shehu transform environment. Phys Scr. 2023;98(8). doi: 10.1088/1402-4896/ace6de

[24] Liaqat MI, Khan A, Alqudah MA, Abdeljawad T. Adapted homotopy perturbation method with Shehu transform for solving conformable fractional nonlinear partial differential equations. World Sci. 2023;31(2). doi: 10.1142/S0218348X23400273

[25] Kapoor M, Shah NA, Saleem S, Weera W. An analytical approach for fractional hyperbolic telegraph equation using Shehu transform in one, two and three dimensions. Mathematics 2022;10(12)

[26] Thange TG, Alure AM. Generalized Shehu transform. J Math Comput Sci 2022;12(27):12–27. doi: 10.28919/jmcs/6922

[27] Jarad F, Abdeljawad T. Generalized fractional derivatives and Laplace transform. Discrete Contin Dyn Syst S 2020;13(3):709–22. doi: 10.3934/dcdss.2020039

[28] Khalaf RF, Belgacem FBM. Extraction of the Laplace, Fourier, and Mellin Transforms from the Sumudu Transform. In: 10th International conference on Mathematical problems in Engineering, Aerospace and Sciences, 2014, pp. 1426–32. doi: 10.1063/1.4907309

[29] Shah K, Junaid M, Ali N. Extraction of Laplace, Sumudu, Fourier and Mellin transform from the natural transform. J Appl Environ Biol Sci. 2015;5(9):1–8.

[30] Chauhan R, Kumar N, Aggarwal S. Dualities between Laplase-Carson transform and some useful integral transform. Int J Innov Technol Explor. 2019;8:1654–9. doi: 10.35940/ijitee.L3163.1081219

[31] Loonker D, Banerji PK. Natural transform and solution of integral equations for distribution spaces. Am J Math Sci. 2014;3(1):2250–3102.

[32] Oldham KB, Spanier J. The fractional calculus: theory and applications of differentiation and integration to arbitrary order. New York and London: Academic Press; 1974.

[33] Belgacem R, Baleanu D, Bokhari A. Shehu transform and applications to Caputo-fractional differential equations. Int J Anal Appl. 2019;17(6):917–27.

[34] Podlubny I. Fractional differential equations. Mathematics in Science and Engineering. New York: Academic Press; 1999.

[35] Bodkhe DS, Panchal SK. On Sumudu transform of fractional derivatives and its applications to fractional differential equations. Asian J Math Comput Res. 2016;11(1):69–77.

[36] Djennadi S, Shawagfeh N, Inc M, Osman MS, Gómez-Aguilar JF, Arqub OA. The Tikhonov regularization method for the inverse source problem of time fractional heat equation in the view of ABC-fractional technique. Phys Scr. 2021;96. doi: 10.1088/1402-4896/ac0867

[37] Arqub OA. Numerical solutions for the Robin time-fractional partial differential equations of heat and fluid flows based on the reproducing kernel algorithm. Int J Numer Methods Heat Fluid Flow. 2018;28(4):828–56.

